# The great escape: patterns of enemy release are not explained by time, space or climate

**DOI:** 10.1098/rspb.2023.1022

**Published:** 2023-08-30

**Authors:** Zoe A. Xirocostas, Jeff Ollerton, Riin Tamme, Begoña Peco, Vincent Lesieur, Eve Slavich, Robert R. Junker, Meelis Pärtel, S. Raghu, Akane Uesugi, Stephen P. Bonser, Giancarlo M. Chiarenza, Mark J. Hovenden, Angela T. Moles

**Affiliations:** ^1^ Evolution and Ecology Research Centre, School of Biological, Earth and Environmental Sciences, UNSW Sydney, New South Wales 2052, Australia; ^2^ Stats Central, Mark Wainwright Analytical Centre, UNSW Sydney, New South Wales 2052, Australia; ^3^ Kunming Institute of Botany, Chinese Academy of Sciences, Kunming, People's Republic of China; ^4^ Faculty of Arts, Science and Technology, University of Northampton, Northampton, UK; ^5^ Institute of Ecology and Earth Sciences, University of Tartu, J. Liivi 2, 50409 Tartu, Estonia; ^6^ Terrestrial Ecology Group (TEG), Department of Ecology, Institute for Biodiversity and Global Change, Universidad Autónoma de Madrid, 28049 Madrid, Spain; ^7^ CSIRO European Laboratory, 830 Avenue du Campus Agropolis, 34980 Montferrier sur Lez, France; ^8^ Evolutionary Ecology of Plants, Department of Biology, University of Marburg, 35043 Marburg, Germany; ^9^ Department of Environment and Biodiversity, University of Salzburg, 5020 Salzburg, Austria; ^10^ CSIRO Health & Biosecurity, Brisbane, Queensland, Australia; ^11^ School of Biological Sciences, Monash University, Clayton, Victoria 3800, Australia; ^12^ Biosciences and Food Technology Division, School of Science, RMIT University, Bundoora, Victoria 3083, Australia; ^13^ Biological Sciences, School of Natural Sciences, University of Tasmania, Hobart, Tasmania 7001, Australia

**Keywords:** enemy release hypothesis, herbivory, introduced species, invasion ecology, biocontrol

## Abstract

When a plant is introduced to a new ecosystem it may escape from some of its coevolved herbivores. Reduced herbivore damage, and the ability of introduced plants to allocate resources from defence to growth and reproduction can increase the success of introduced species. This mechanism is known as enemy release and is known to occur in some species and situations, but not in others. Understanding the conditions under which enemy release is most likely to occur is important, as this will help us to identify which species and habitats may be most at risk of invasion. We compared *in situ* measurements of herbivory on 16 plant species at 12 locations within their native European and introduced Australian ranges to quantify their level of enemy release and understand the relationship between enemy release and time, space and climate. Overall, plants experienced approximately seven times more herbivore damage in their native range than in their introduced range. We found no evidence that enemy release was related to time since introduction, introduced range size, temperature, precipitation, humidity or elevation. From here, we can explore whether traits, such as leaf defences or phylogenetic relatedness to neighbouring plants, are stronger indicators of enemy release across species.

## 1. Introduction

Herbivores are the bane of almost any plant's existence and can severely limit individual fitness and population growth [1–5]. In most natural ecosystems, plants and their herbivores have co-evolved over millions of years, with plants gaining protective traits to reduce damage, and herbivores adapting to overcome plant defences [6–8]. As such, interactions between plants and herbivores can become unique to the ecosystems they naturally inhabit [9]. So, when a plant is introduced to a new ecosystem it may be freed from the constraints of the herbivores that once restricted it in its native range [10]. This mechanism is referred to as enemy release [10–13].

Escaping from the enemies (e.g. herbivores and/or pathogens) that co-evolved with a plant species in its native range can be a major contributor to a species' success in an introduced range, as they may allocate resources to growth and reproduction instead of repair and defence [10]. However, studies suggest that only about half of introduced species actually experience enemy release [10,12,14–17]. Most of our understanding of enemy release tends to focus on case studies of one or a small number of species, with relatively few examples of field comparisons across multiple species and locations [18–20]. The limited taxonomic scope of most previous studies means that we have no empirical evidence about the spatial, temporal and climatic circumstances that might allow us to predict whether a particular introduced plant species is likely to experience enemy release. Our study addresses this knowledge gap using a biogeographic approach to quantify the factors contributing to successful enemy release in a broad range of plant species in multiple, diverse locations within their native and introduced ranges.

We first ask whether the amount of herbivore damage our study species receive differs between their native and introduced ranges. Answering this question allows us to understand which plants are experiencing enemy release and the magnitude to which they are affected, allowing us to explore further questions on the factors contributing to enemy release. We hypothesize that plants in the introduced range will suffer less herbivore damage overall, as they are more likely to have escaped their enemies according to the enemy release hypothesis [10,11].

We then test a range of hypotheses that aim to better predict when and where enemy release is most likely to occur.

Our first prediction is that the magnitude of enemy release plant species experience will decrease with time since introduction. Native herbivores, especially those with specialized interactions, usually prefer to feed from the native plants they have co-evolved with, and can struggle to tolerate the defensive mechanisms employed by invasives [21,22]. Yet as time passes, some introduced species may eventually accumulate ‘enemies' as herbivores switch feeding between native and introduced hosts, as shown by Rodríguez *et al*. [21] in a case study of *Acacia dealbata* and *Carpobrotus edulis* invasions on the Iberian peninsula. However, a study, spanning 35 species, showed no effect of time since introduction in relation to a plant's degree of herbivory [23]. A meta-analysis found that enemy release is higher in species that were introduced more recently (less than 50 years ago) and lower in plants that had established earlier (50–200 years ago), with herbivory levels similar to conspecifics in their native range [14]. Our study extends and complements these previous findings and is the first to account for variation in enemy release across multiple species and sites within the native and introduced ranges.

Subsequently, we ask whether the degree to which species experience enemy release is negatively correlated with their introduced range size. According to the species–area relationship, larger areas can foster a greater diversity of organisms in comparison to smaller fragments and studies have shown that arthropod diversity is best predicted by the range size of host plants [12,24]. However, no studies have previously tested whether a relationship between range size and enemy release exists. As plant species with smaller range sizes are less likely to encounter and accumulate a diversity of herbivores than those with larger range sizes, we predict that species with smaller introduced range sizes are more likely to experience stronger enemy release.

Finally, we ask whether enemy release is correlated with the climate or elevation of the introduced sites they occupy. As ectotherms, invertebrate herbivores' metabolism and rate of consumption are regulated by their external environment, and rise with increasing temperature [25–27]. Patterns with water availability are less clear, with some evidence that leaf damage increases with precipitation [28,29], but other evidence that relative humidity is negatively correlated with herbivory [30]. The negative relationship with relative humidity could be explained by humidity's inversely proportional relationship to temperature, as air becomes drier as temperature increases, which, in turn, increases the rate of herbivory. Invertebrate presence and leaf damage are also lower at higher altitudes, possibly due to lower temperatures and resource availability [31,32]. We therefore hypothesize that enemy release will be negatively correlated with temperature and precipitation, and positively correlated with humidity and elevation.

In summary, we predict:

1. Overall, plants will experience more herbivore damage in their native range than in their introduced range.
2. Enemy release will decrease with time since introduction.
3. Enemy release will decrease with the size of the invaded range.
4. Enemy release will decrease with increasing temperature and precipitation.
5. Enemy release will increase with humidity and elevation.

## 2. Material and methods

### (a) Data collection

**Figure 1.**
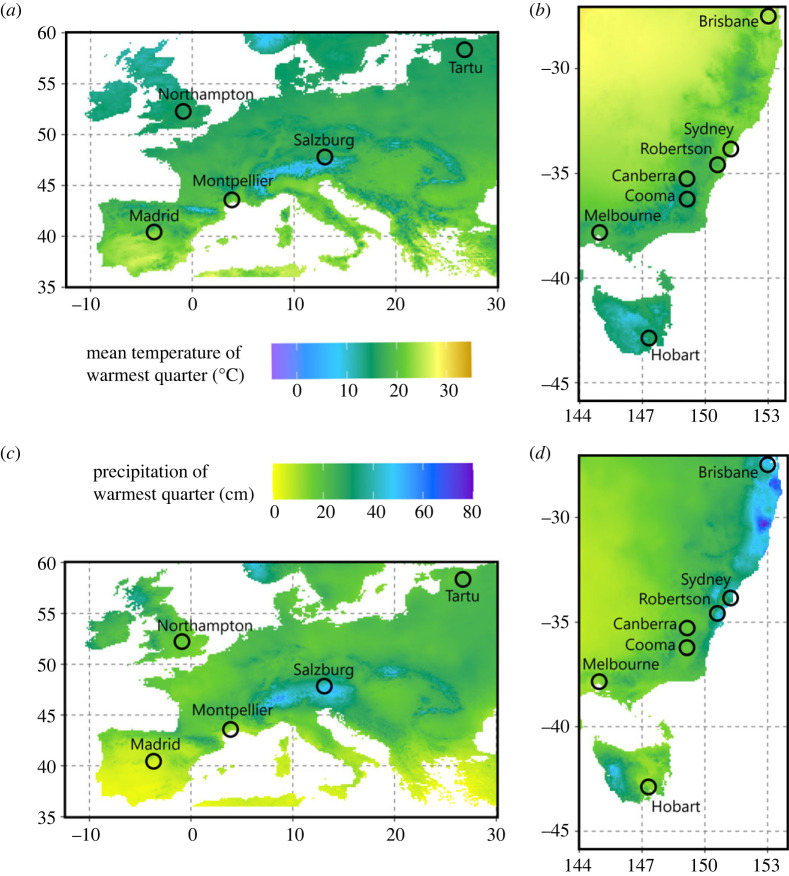
Maps of sampling sites in (*a*,*c*) Europe (native range) and (*b*,*d*) Australia (introduced range). Sites in Europe include Madrid (Spain), Montpellier (France), Salzburg (Austria), Northampton (United Kingdom) and Tartu (Estonia). Sites in Australia include Hobart (Tasmania), Melbourne (Victoria), Cooma (New South Wales), Canberra (Australian Capital Territory), Robertson (New South Wales), Sydney (New South Wales) and Brisbane (Queensland). Maps are shaded according to (*a*,*b*) mean temperature of the warmest quarter and (*c*,*d*) total precipitation of the warmest quarter from WorldClim version 2.1 climate data for 1970–2000 [33].

To determine whether introduced vascular plant species are experiencing enemy release in Australia, we measured leaf damage at 5 sites in the native range and 7 sites in the introduced range of 16 plant species (figure 1). We incorporated data from ecologically diverse locations (i.e. the dry, warm mountainous region of northern Madrid to the cool, damp meadows of the English midlands) within each range, to better reflect the variation in herbivory that plants can receive across different habitats/populations. We confirmed each species' status as either native to Europe, or introduced to Australia, from the literature.

We chose our target species based on three main criteria whereby each species must:

1) Have a widespread presence in both Europe (as a native plant) and southeastern Australia (as an introduced plant).
2) Not actively be managed by biocontrol agents in Australia (because biocontrol agents work by countering enemy release).

This yielded a list of over 25 plant species eligible for inclusion in our study. However, despite our best efforts in the field, some species could not be located and measured at least once in the native range and once in the introduced range. Our third criteria was thus that species were measured in at least one site across both ranges (native and introduced). Our final dataset includes measurements from 16 herbaceous plant species (15 eudicots and 1 monocot) belonging to 14 families and 11 orders (electronic supplementary material, appendix S1). Of these species, six (*Convolvulus arvensis*, *Hypericum perforatum*, *Leucanthemum vulgare*, *Parietaria judaica*, *Rumex acetosella* and *Verbascum thapsus*) are listed as invasive by Weeds Australia (https://weeds.org.au/).

When choosing our study sites, we prioritized maximizing the latitudinal range and landscape diversity in each range. Target species presence was also factored into site choice as we preferred to visit places that would increase our sampling potential. We used online databases such as the Global Biodiversity Information Facility (gbif.org) and the Atlas of Living Australia (ala.org.au) to assess target species presence prior to choosing our site locations. Not all study species were present at each site (i.e. city or region where sampling took place) (electronic supplementary material, appendix S2).

At each site, we aimed to measure foliar herbivory on 10 leaves of at least 12 individuals per species. A random number generator and compass were used to determine the observer's orientation, and we measured the first individual we encountered in this direction. We repeated this process until 12 plants were measured. We distinguished individuals by ensuring they were spaced at least 2 m apart, with clonally spreading species requiring at least 5 m distance; however, most individuals were spaced further than this. We began measuring from the first fully expanded leaf on the highest branch and continued towards the base of the stem. Where there were fewer than 10 leaves on a branch, we continued to measure on the branch/es directly below until 10 measurements were recorded. Where there were fewer than 10 leaves per individual, we compensated by measuring more individuals until we reached a similar number of measured leaves. Species with compound leaves (e.g. *Trifolium repens* and *Lotus corniculatus*) had their herbivory measured per leaflet (10 leaflets of 12 individuals) in a clockwise direction from the petiole. The herbivory examined in this study is ectophagy and does not consider the identity of the herbivores or their functional interactions.

Herbivory measurements were calculated as a percentage of removed or damaged leaf tissue, including the lamina and petiole. Types of herbivory we encountered include chewing, mining, galling, skeletonizing and sucking. Visual estimates were used to assess herbivory on a scale of 0–100%, by mentally dividing the leaf into four equal quadrants and visualizing the damage all together in one section [34]. We chose to estimate leaf damage visually as it only takes approximately 10 s to measure each leaf, allowing us to notably increase our sample size and perform all observations in the field [35–37]. All visual estimates of herbivory were conducted by the lead author (Z.A.X.) after being trained to measure herbivory on leaf images with known damage. Assessor accuracy was assessed twice in the field (once in Europe and once in Australia) by visually estimating a subsample of leaves and then digitally analysing their amount of leaf damage using ImageJ. All visually assessed estimates were within 1% accuracy of the digital measurements. Field observations took place in the peak growing seasons of 2019, from May to July in Europe and between September and November in Australia.

To assess whether enemy release is related to plant species' time since introduction, we compiled data on species' year of introduction to Australia from the literature. The literature reports initial occurrences of species introductions (or estimates thereof) to the continent of Australia but does not account for multiple introductions of a species to varying regions. However, as we are testing this relationship on the macro-scale, coarser records are sufficient, as any pattern arising from data with greater uncertainty would only strengthen its support for a relationship. For each target species, we searched two online databases, the Atlas of Living Australia (ala.org.au) and the Web of Science, to determine the year of their earliest known occurrence in Australia. For the Atlas of Living Australia, we simply searched each species by scientific name to access their earliest recorded occurrence in Australia. For the Web of Science, we used keywords such as ‘year', ‘introduc*' and ‘Australia' accompanied by scientific name. We calculated time since introduction by subtracting species' year of introduction from the year herbivory observations took place (2019).

To understand whether enemy release is associated with plant species' introduced range size, we gathered range size data from the Atlas of Living Australia's spatial portal (spatial.ala.org.au; accessed 22 June 2021; electronic supplementary material, appendix S3). We chose ‘area of occupancy' as a metric to assess our species' geographical spread. We added each species, separately, into the spatial portal (restricting records to only those that were spatially valid and within Australia) and used the ‘calculate AOO and EOO' function (with a grid resolution of 0.05 decimal degrees and alpha hull of 2) to attain the area of occupancy (km^2^), which we hereby refer to as range size for introduced populations.

To understand whether enemy release is associated with climate and elevation we downloaded data from:

1. WorldClim v2.1 at 2.5 minute resolution [33] for mean annual temperature, annual precipitation, mean temperature of the warmest quarter and precipitation of the warmest quarter in the native and introduced ranges, and elevation in the native range only. Mean annual temperature and annual precipitation were chosen as they are meaningful predictors for plant growth, insect activity and herbivore consumption [38,39]. We also considered the mean temperature of the warmest quarter and total precipitation of the warmest quarter as this is widely regarded as the peak season for plant growth and herbivore consumption [26,40].
2. The 3 s STRM Derived Digital Elevation Model (DEM) v1.0 [41] for elevation in the introduced range.
3. The Australian Bureau of Meteorology's gridded dataset for mean annual relative humidity at 15.00 at 0.1° resolution (available from http://www.bom.gov.au/web01/ncc/www/climatology/relative-humidity/rh15/rh15an.zip) for relative humidity in the introduced range. We used relative humidity at 15.00 instead of 21.00, as humidity is higher in the mornings in most locations which is not representative of the humidity experienced by plants/herbivores for most of the day (US Department of Commerce).
4. New *et al*. [43] for relative humidity in the native range. These values were averaged across 12 months and rasterized at a resolution of 0.25°.

All values for abiotic variables were extracted from the aforementioned datasets using the specific coordinates where each species was located at each site, and running a nearest-neighbour interpolation in QGIS v3.24 [44].

### (b) Data analysis

All statistical analyses were performed in R version 4.2.0 [45].

To understand the direction and magnitude of enemy release, we ran generalized linear mixed models using Template Model Builder [46]. We used the amount of herbivory plants received as our response variable, range (introduced or native) as our predictor variable, and included random effects terms for site, species and individual. As our data contained many zeros, we used the Tweedie family with log-link function to fit our model. The coefficient for range represents the ratio of herbivory in the native to herbivory in the introduced range, on a log scale (i.e. it represents enemy release). Our data did not require any prior transformation as they satisfied all model assumptions.

Next, we tested for an association between enemy release and time since introduction by performing a linear model with the *lm* function in base R [45]. Our response variable was enemy release (using model coefficients for each species from our first herbivory model, i.e. ln(mean herbivory in native range/mean herbivory in introduced range)) and our predictor variable was time since introduction. We used the species' coefficients from our first model as they accounted for variance in herbivory between individual plants and sites. Our model was weighted by the inverse standard error of our original herbivory model coefficients. We used a similar weighted model to quantify the relationship between enemy release and plants' range size in Australia. Enemy release, using previous model coefficients again, was our response variable and log_10_-range size was our predictor variable.

After analysing the last two models, we decided to test whether time since introduction influenced the amount of area that species would end up occupying in their introduced range. To do this, we ran a linear model with our predictor variable as species' time since introduction and response variable as introduced range size using the *lm* function in base R [45].

**Figure 2.**
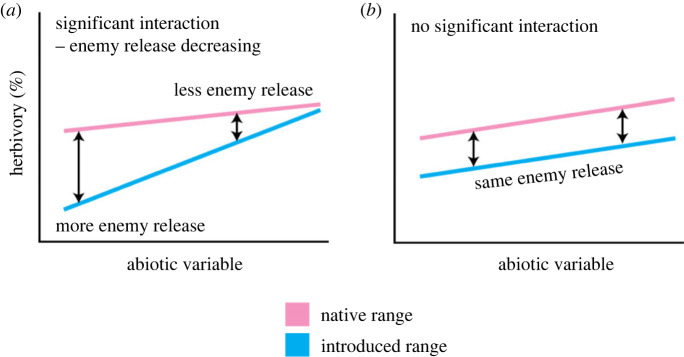
Graphical illustration of potential ANCOVA outcomes. (*a*) A significant interaction indicates that the slope of the relationship between the abiotic variable and herbivory differs between the native and introduced range. For example, decreasing distance between lines for species in the native and introduced ranges indicates decreasing enemy release (i.e. decreasing ratio of herbivory in native/introduced ranges) as the abiotic factor increases. (*b*) Similar distance between lines for species in native and introduced ranges indicates similar enemy release regardless of the abiotic factor increasing (i.e. similar ratio of herbivory in native/introduced ranges).

Finally, we asked whether climatic conditions and elevation of sites affect the magnitude of enemy release plants experience. We did this by calculating a weighted average of herbivory for each species at each site in the introduced and native ranges. First, we calculated the arithmetic mean (after adding 0.005 to each observed value to avoid zeros (as in [47])) of log-transformed herbivory for each individual, per site. Next, we calculated site-level herbivore damage for each species as the exponent of arithmetic mean herbivory across the individual plants at each site. We then performed separate ANCOVAs using mean herbivory (per species per site) as our response variable, an interaction term encompassing range and an abiotic factor as our explanatory variable, and site and species as random effects terms. To meet model assumptions, we square-root transformed mean herbivory prior to analyses. For these analyses, a significant interaction could indicate enemy release as either decreasing (figure 2*a*) or increasing with the abiotic variable. A non-significant interaction indicates no relationship between enemy release and the abiotic variable (figure 2*b*).

## 3. Results

**Figure 3.**
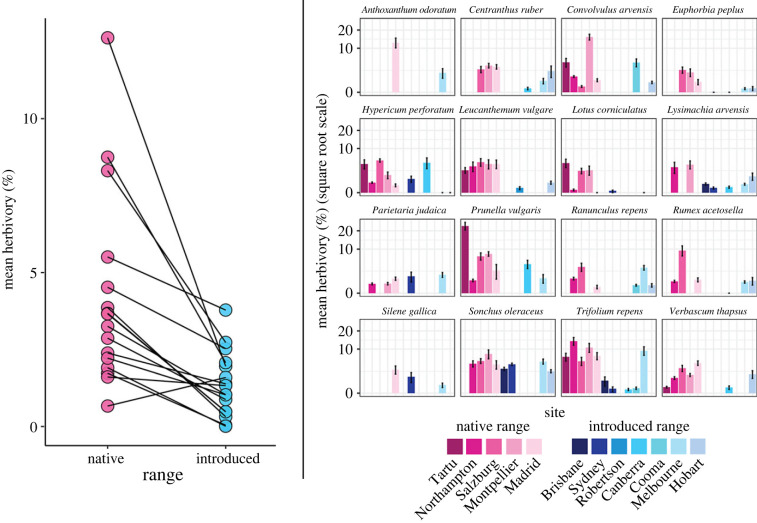
[Left] Comparison of mean herbivory between native (pink) and introduced (blue) ranges for each species site-weighted average herbivory in native and introduced ranges. [Right] Variation in mean herbivory across sites in the native and introduced ranges for each target species. Bars represent means ± s.e.

After conducting fieldwork across twelve sites, six countries and two continents, we had recorded 11 600 separate visual estimations of herbivory (6142 in the native range and 5458 in the introduced range) for 16 plant species. Consistent with the enemy release hypothesis, we found that overall, our species experience greater herbivory in their native range than in their introduced range (figure 3; *p* < 0.0001) with an effect size of 1.88 (95% confidence interval from 1.10 to 2.66). In biological terms, this means that plants in their native range are suffering from 6.55 times more leaf damage than conspecifics in their introduced range. Individually, all 16 species tended towards greater herbivory in the native range, with half being statistically significant (95% confidence intervals did not overlap zero).

**Figure 4.**
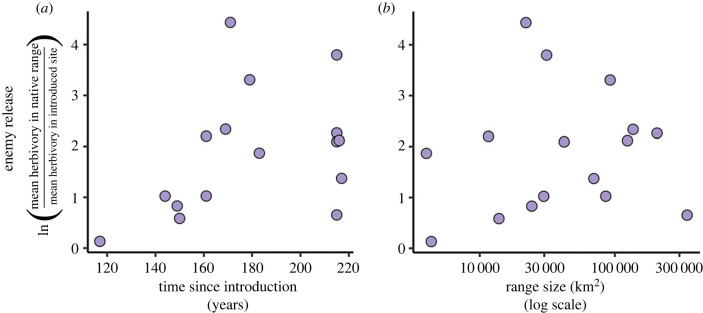
The relationship between plants' (*a*) time since introduction (*p* = 0.13) and (*b*) range size (*p* = 0.66) in Australia to their degree of enemy release. Range size is calculated as the sum of grid squares (at 0.05 decimal degree resolution) that are occupied by a species. Each point represents a target species (*n* = 16). Neither model showed evidence for an association between variables.

Contrary to our prediction, we found no evidence for a correlation between species' degree of enemy release and time since introduction (figure 4*a*; *p* = 0.13, adjusted *R*^2^ = 0.10, *F*_1, 14_ = 2.60).

There was no significant relationship between species' degree of enemy release and the amount of introduced area they currently occupy (figure 4*b*; *p* = 0.66, adjusted *R*^2^ = −0.06, *F*_1, 14_ = 0.20).

**Figure 5.**
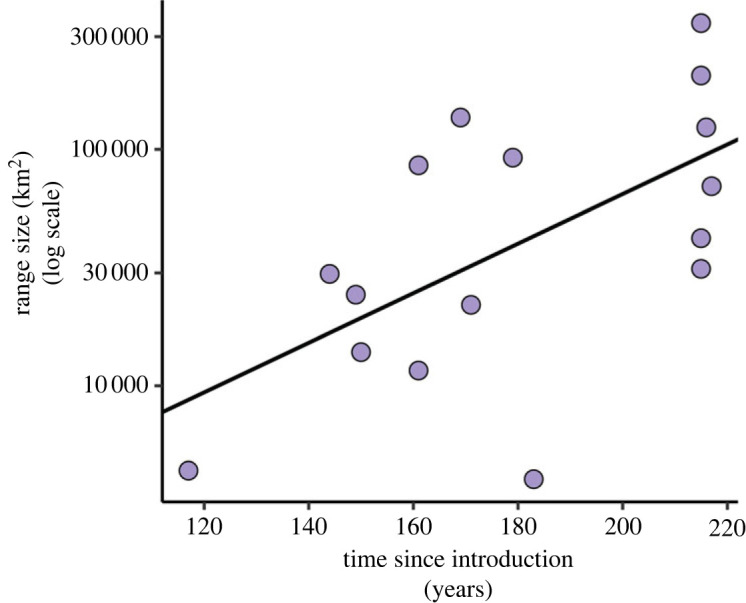
The relationship between species' time since introduction and the amount of introduced area they occupy (*p* = 0.01, adjusted *R*^2^ = 0.32, *F*_1, 14_ = 7.91). Each point represents one species.

Although it was not one of our initial hypotheses, we did find a positive relationship between species' range size and time since introduction (figure 5; *p* = 0.01, adjusted *R*^2^ = 0.32, *F*_1, 14_ = 7.91).

Counter to our predictions, we found no evidence for an interaction between range and any abiotic variable, in relation to the amount of herbivory plants receive (i.e. plants experience similar enemy release regardless of climate/elevation levels) (table 1). That is, none of our abiotic variables helped to predict when introduced species experience enemy release.

**Table 1.** Model outputs showing no significant interaction (*p* > 0.05) between any of our abiotic variables with native/introduced range.

abiotic variable in model	denominator degrees of freedom	*F* value	*p*-value of interaction between range (native versus introduced) and the abiotic variable
annual precipitation	16.51	1.10	0.31
mean annual temperature	13.35	0.04	0.84
precipitation of the warmest quarter	15.78	1.03	0.32
mean temperature of the warmest quarter	10.86	0.17	0.68
humidity	8.56	0.53	0.49
elevation	14.40	0.003	0.95

## 4. Discussion

We did not find that time, space or climate are related to the magnitude of enemy release plants experience in their introduced range (figure 4 and table 1). This null result is important, as it suggests that enemy release, one of the major factors underpinning the success of introduced species, cannot be predicted by the abiotic factors of plants' novel environments. Our study did not encompass the full suite of the world's ecosystems but did include sites ranging in mean annual temperature from 5.3°C to 20.4°C, in total annual precipitation from 40.4 cm to 150 cm, and in elevation from 2 m to 1353 m. Our findings suggest that biocontrol, the flip-side of enemy release, should be equally likely to succeed or fail independent from the ecosystems they inhabit.

Knowing the ecological context behind a species invasion is a crucial step to implementing practices to hinder the spread of introduced species [48]. In most cases, classic biological control is employed to target problematic invasive species with the aim to slow or decrease their population growth with minimal impact on surrounding native species [49]. These reductions in invasive populations can be achieved by releasing known above- or below-ground herbivores, predators or pathogens, that are native to the same areas as the invasive species, as controlling agents [50]. There are many successful examples of biocontrol around the world [51–53] and meta-analyses by Stiling & Cornelissen [55] found that biocontrols can reduce the biomass and reproductive output of weeds by over 80%. But not all instances of biocontrol succeed. Failed attempts at biologically controlling invasive plants have been recorded globally [50,56]. Plant species that have been identified as being released from their enemies should theoretically have the highest chance of successful management with biological control, as enemy release likely contributes to their successful invasion [57]. However, our study implies that biocontrol is equally likely to be effective under a range of abiotic conditions, and regardless of introduced species' time since introduction into a novel range or range size.

There is much more variation in plants’ potential to encounter enemies in the introduced range than originally expected, which might help to explain the lack of correlation between enemy release and time since introduction and introduced range size. For example, a plant that has recently established in a highly disturbed area with a high diversity of other introduced species may be more likely to encounter compatible herbivores than plants that have established earlier in a more stable habitat with fewer introduced species. Similarly, a non-native species occupying a smaller area of space, with more generalist herbivores, may experience greater herbivore pressure than plants occupying a more expansive patch of land that houses fewer generalist herbivores.

We did find a relationship between introduced species' geographical spread and the amount of time they have had to establish themselves in their new range (figure 5). This finding corroborates many preceding studies in invasion ecology that have also shown that distribution in the non-native range is strongly correlated with time since introduction and demonstrates that our sampling effort is rigorous enough to detect this pattern [58–64]. Remarkably, some introduced plants have been found to colonize local areas at rates of up to 370 m per year and long distances at up to 167 km yr^−1^ [65].

The lack of a significant relationship between enemy release and abiotic factors such as climate and elevation could arise from herbivory not being explained by these variables (see electronic supplementary material, appendix S4). Some studies have shown no significant relationship between herbivory and temperature or precipitation [66,67], while others have found that herbivory increases [38,68–70] or decreases with temperature or precipitation [71–73], and others have found mixed results [74,75]. However, even where significant positive correlations have been detected, they tend to have *R*^2^ values below 0.3 [39,76]. Empirical evidence for an effect of humidity and elevation on herbivory is much scarcer, and available research does not explore these relationships at global scales, or across multiple species [30,31].

While we have collected data from a broad range of species from varying locations in their native and introduced ranges, we acknowledge that our study may be limited by the fact it compares a minor subset of the world's introduced species, in one continent of their non-native range. Ultimately, we present data from 5 sites in the native range and 7 sites in the introduced range, for 16 species. While we selected these study sites to maximize the range of climatic conditions sampled, and our sample size was sufficient to detect patterns in range size and time since introduction, it is possible that the lack of significant relationships between climate variables and enemy release is partly attributable to our sample size. Thus, our results should be considered as a first step to eventually uncovering global trends in spatial, temporal and climatic patterns of enemy release. We hope that future studies will replicate ours using different species, in different locations, to expand our knowledge of how this invasion mechanism works in more extreme ecosystems and other continents.

Our finding that enemy release is not directly related to time since introduction, range size or climate is new and valuable information that may influence the trajectory of our use of biocontrols, with the potential to prompt their implementation in new areas and on new target species. We hope this study will trigger future research to explore more factors, such as herbivore specialization or defensive traits, that may affect species success in new ranges, so we may find clearer answers relating to the spread of introduced plants. If we are to conserve and protect Earth's natural ecosystems, of which almost all have been considered invaded by non-native species, then enhancing our understanding of the mechanisms affecting these invasions is critical [77].

## Data Availability

Code and data associated with this study are publicly available on Figshare via the following links: https://doi.org/10.6084/m9.figshare.23639844.v1, https://doi.org/10.6084/m9.figshare.23639859.v1. The data are provided in electronic supplementary material [78].
